# Impact of Reclassification of Oncocytic and Follicular Thyroid Carcinoma by the 2022 WHO Classification

**DOI:** 10.1210/clinem/dgae581

**Published:** 2024-08-21

**Authors:** Merel T Stegenga, Lindsey Oudijk, Evert F S van Velsen, Robin P Peeters, Marco Medici, Frederik A Verburg, Tessa M van Ginhoven, Folkert J van Kemenade, W Edward Visser

**Affiliations:** Erasmus MC Academic Center for Thyroid Diseases, Department of Internal Medicine, Erasmus Medical Center, Rotterdam, 3015GDThe Netherlands; Erasmus MC Academic Center for Thyroid Diseases, Department of Pathology, Erasmus Medical Center, Rotterdam, 3015GDThe Netherlands; Erasmus MC Academic Center for Thyroid Diseases, Department of Internal Medicine, Erasmus Medical Center, Rotterdam, 3015GDThe Netherlands; Erasmus MC Bone Center, Department of Internal Medicine, Erasmus Medical Center, Rotterdam, 3015GDThe Netherlands; Erasmus MC Academic Center for Thyroid Diseases, Department of Internal Medicine, Erasmus Medical Center, Rotterdam, 3015GDThe Netherlands; Erasmus MC Academic Center for Thyroid Diseases, Department of Internal Medicine, Erasmus Medical Center, Rotterdam, 3015GDThe Netherlands; Erasmus MC Academic Center for Thyroid Diseases, Department of Radiology and Nuclear Medicine, Erasmus Medical Center, Rotterdam, 3015GDThe Netherlands; Erasmus MC Academic Center for Thyroid Disease, Department of Surgery, Erasmus Medical Center, Rotterdam, 3015GDThe Netherlands; Erasmus MC Academic Center for Thyroid Diseases, Department of Pathology, Erasmus Medical Center, Rotterdam, 3015GDThe Netherlands; Erasmus MC Academic Center for Thyroid Diseases, Department of Internal Medicine, Erasmus Medical Center, Rotterdam, 3015GDThe Netherlands

**Keywords:** thyroid neoplasms, follicular thyroid carcinoma, oncocytic thyroid carcinoma, pathology, survival, WHO Classification

## Abstract

**Background:**

The 2022 WHO Classification categorizes oncocytic (OTC) and follicular thyroid carcinoma (FTC), based on the degree of capsular and vascular invasion, into minimally invasive (MI), encapsulated angio-invasive (EA), and widely invasive tumors (WI). While associations with clinical outcomes have been studied extensively in FTC, robust clinical data are lacking for OTC. We aimed to investigate the impact of the reclassification of OTC and FTC by the 2022 WHO Classification on clinical outcomes.

**Methods:**

All adult OTC and FTC patients treated at the Erasmus MC (the Netherlands) between 2000 and 2016 were retrospectively included. All tumors were extensively revised by 2 independent pathologists, facilitated by Palga: Dutch Pathology Databank. Kaplan-Meier curves were used to study the association of the 2004 and 2022 WHO Classification with overall survival, disease-specific survival (DSS), recurrence-free survival, and radioactive iodine (RAI)-refractory disease.

**Results:**

Among 52 OTC and 89 FTC patients, 15 (28.8%) OTC and 34 (38.2%) FTC tumors were reclassified as EAOTC or EAFTC. The 2022 WHO Classification substantially improved risk stratification in both subtypes for DSS, compared to the 2004 edition. Ten-year DSS rates were 100% for MIOTC, 92.9% for EAOTC, and 56.5% for WIOTC, compared to 100% (MIOTC) and 64.2% (WIOTC) following the 2004 WHO Classification. For FTC and RAI-refractory disease, similar trends were observed.

**Conclusion:**

Classification of OTC and FTC into 3 subcategories as defined by the 2022 WHO Classification substantially improves discrimination between low-, intermediate-, and high-risk patients, especially for DSS and RAI-refractory disease.

Over the past 2 decades, the incidence of differentiated thyroid carcinoma (DTC) has steadily been rising (1, 2). This rise is primarily due to the increasing incidence of 1 particular subtype of DTC, papillary thyroid carcinoma (PTC), while the incidence of the other subtypes has remained stable during this period (3, 4). Follicular thyroid carcinoma (FTC) is the second most common DTC, but with a prevalence of 5-10%, it is still relatively rare (5, 6). Oncocytic thyroid carcinoma (OTC), previously known as Hürthle cell carcinoma, is even less common with estimates around 3-5% of DTCs (7). In general, treatment for both FTC and OTC consists of a total thyroidectomy and radioactive iodine (RAI) with a 10-year disease-specific survival (DSS) of 80-94% (8-10).

Until 2017, OTC was considered a subtype of FTC due to assumed similarities in clinical and histological characteristics, such as capsular (CI) and vascular invasion (VI) (11). In 2017, however, the 4th edition of the WHO Classification of Tumours of Endocrine Organs made a definitive distinction between FTC and OTC, categorizing both as separate disease entities (12). This was primarily based on the distinct genetic landscape of OTC, but also on growing evidence that OTC behaves significantly differently than its FTC counterpart from a clinical point of view (13-15). Specifically, regarding RAI-avidity and DSS, OTC seems to be less responsive to the current treatment strategies than FTC, emphasizing the need for more research that distinguishes OTC from FTC (16-18).

Another important change with the 4th edition of the WHO Classification is the categorization of FTC into 3 subtypes instead of 2, introducing encapsulated angio-invasive FTC (EAFTC) (12). In the 2004 WHO Classification, FTC was divided into minimally invasive (MI) and widely invasive (WI), based on the extent of CI and VI (19). However, with the 2017 edition, a distinction was made between encapsulated tumors with or without VI, as the presence and extent of VI were strongly associated with survival in FTC patients (20-22). In 2022, the 5th edition of the WHO Classification was released, in which this categorization into 3 subtypes was also adopted for OTC, now distinguishing MIOTC, EAOTC, and WIOTC. However, while this new classification has been studied in FTC (9, 20, 21, 23-25), for OTC, robust clinical data has been lacking. Furthermore, no direct comparison of the new 2022 WHO Classification with the 2004 WHO Classification has been undertaken before.

Therefore, we aimed to investigate the impact of the reclassification of OTC and FTC according to the 2022 WHO Classification categories (ie, MI, EA and WI) on relevant clinical outcomes, facilitated by an extensive histopathological review.

## Methods

### Study Population

All adult patients diagnosed and/or treated for OTC and FTC at the Erasmus University Medical Center in Rotterdam (the Netherlands) between 2000 and 2016 were retrospectively included. Patients were excluded from the analysis if histological slides were not available or in case of pT0. Demographic information and treatment data were obtained from electronic patient records, and included sex, age at diagnosis, extent of surgery, lymph node dissection, and RAI therapy. Disease staging was done according to the 8th AJCC/TNM Staging System (26). Follow-up time was defined as the time from diagnosis to the last moment of contact, end-of-study (31 December 2020) or death, whichever occurred first. The Medical Ethics Review Committee of the Erasmus Medical Center provided a waiver for approval, and no informed consent was required (filed under MEC 2018-1195).

### Pathology Revision

After retrieval of histological slides through the Dutch National Tissue Bank portal via the Dutch Nationwide Pathology Databank (Palga) (27), 2 board-certified pathologists with a special interest and experience in thyroid carcinoma (F.K. and L.O.) independently examined all available slides systematically, blinded to outcomes. In case of discordant cases (up to 11%), the pathologists revised the material together to achieve consensus. In 136 cases (96.5%), the entire tumor was enclosed for review with a mean of 15 ± 8 slides (mean tumor size 43.0 ± 24.0 mm)). In the remaining 5 cases, the mean number of slides was 4.8 ± 1.3 slides with an average tumor diameter of 35.0 ± 11.2 mm, which was considered adequate sampling.

**Figure 1.**
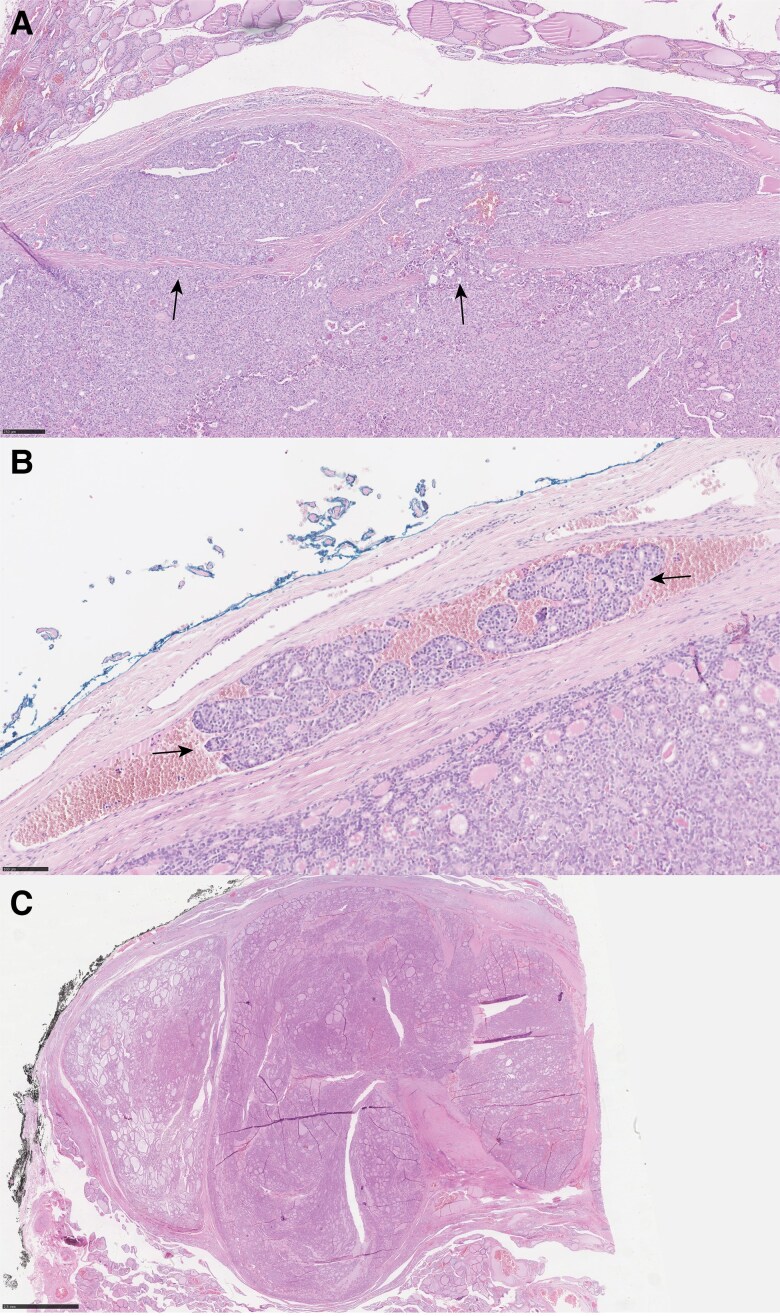
Minimally invasive (MI), encapsulated angio-invasive (EA), and widely invasive carcinoma (WI). A, MIFTC with a focus of capsular invasion; B, EAFTC with a focus of vascular invasion; C, WIOTC with extensive capsular invasion.

Tumors were classified as OTC if more than 75% of the tumor cells showed oncocytic metaplasia and lacked nuclear features of PTC. Tumors with poorly differentiated or anaplastic morphology were excluded, as well as (follicular variant of) PTC and adenomas of any type. Each tumor was assessed and diagnosed according to the definitions of the 2004 and 2022 WHO Classification of Endocrine and Neuroendocrine Tumours (11, 12, 28). Following the 2004 WHO Classification, MI tumors exhibited limited capsular (CI) and vascular invasion (VI), whereas WI tumors were defined as widespread growth into (extra-)thyroidal tissue (19). Based on the 2022 WHO Classification, encapsulated tumors with only CI and no VI were diagnosed as MI (see Fig. 1A). Encapsulated lesions with VI and with or without CI were classified as EAFTC or EAOTC (see Fig. 1B), and WI tumors showed extensive growth into the thyroid or adjacent thyroid tissue, with no or partial encapsulation, often with a multinodular pattern (see Fig. 1C) (29). CI was defined as full-thickness penetration of the capsule, in accordance with the definition used in the WHO Classification (29). The number of CI foci was counted, ensuring that each focus was spatially apart to prevent counting 1 focus multiple times (for example, if a focus of CI was seen in consecutive sections, it was counted as 1 focus) Foci of pseudo-invasion as a consequence of fine needle aspiration or biopsy were not categorized as CI. VI was only diagnosed when the tumor invaded vessels within the tumor capsule or beyond with intravascular tumor attached to the vessel wall or associated with fibrin or covered by endothelium (29). Furthermore, tumor size, presence of tumor capsule (eg, only if entirely circumscribed), extra-thyroidal extension, CI with the number of foci (in case of encapsulated tumors), and VI with number of foci were documented. Focal CI or VI was defined as 1 to 3 foci, whereas ≥ 4 foci were considered extensive CI or VI.

### Clinical Outcomes

Survival time was recorded for each patient and was defined as the total time between the date of diagnosis to the date of death due to any cause (overall survival [OS]) or thyroid carcinoma (disease-specific survival [DSS]). Recurrence-free survival (RFS) was assessed in patients with no evidence of disease after initial therapy and/or during follow-up for at least 1 year and was defined as the time from diagnosis to new biochemical or structural disease. RAI-refractory disease was defined as meeting either 1 of the following definitions, as defined by the 2015 ATA Guidelines: (i) no uptake after the first whole-body scan; (ii) loss of RAI-uptake; (iii) uptake in some but not all lesions; or (iv) progression of disease despite sufficient RAI dosage (30). All other patients were considered to have RAI-avid disease.

### Statistical Analysis

All cohort and tumor characteristics were reported as means with SD, medians with interquartile range (IQR), or absolute numbers with percentages. Patient characteristics were compared with the Student *t* test or χ^2^-test. Survival analysis was performed using Kaplan-Meier curves for OS, DSS, and RFS, and for the incidence of RAI-refractory disease, the cumulative incidence function was used. The log-rank test was performed if the proportional hazard assumption was met, and otherwise, the Gehan-Breslow-Wilcoxon test was done (31). A *P* value < .05 was considered significant. All statistical analyses were performed in R (version 4.2.2) and GraphPad Prism version 9.0 (GraphPad Software, San Diego, CA, USA).

## Results

### Study Population

A total of 141 patients were included, among whom 52 had OTC and 89 FTC (see Table 1). OTC patients were older at diagnosis (61.7 years vs 51.7 years; *P* < .001) and more often male (50% vs 23.6%; *P* = .002) than FTC patients. All patients received total thyroidectomy and at least 1 RAI therapy, in accordance with past and current Dutch guidelines (32). The median follow-up time was 8.5 years [IQR, 5.0-11.4], during which more OTC patients developed RAI-refractory disease (42.3% vs 15.7%; *P* = .002). Furthermore, more OTC patients died due to thyroid carcinoma than FTC patients (26.9% vs 6.7%; *P* = .001).

**Table 1.** Study cohort characteristics

	OTC (n = 52)	FTC (n = 89)	*P* value*^b^*
Male sex (%)	26 (50.0)	21 (23.6)	.002
Age at diagnosis (years)	61.70 (12.13)	51.66 (17.96)	<.001
pT-stage*^a^* (%)			.299
pT1	6 (11.5)	16 (18.2)	
pT2	15 (28.8)	34 (38.6)	
pT3	26 (50.0)	31 (35.2)	
pT4	5 (9.6)	7 (8.0)	
Lymph nodes metastasis at presentation (%)			.435
Present	7 (13.5)	7 (7.9)	
Absent	45 (86.5)	81 (91.0)	
Distant metastasis at presentation (%)			.898
Present	10 (19.2)	15 (16.9)	
Absent	42 (80.8)	74 (83.1)	
Neck dissection (%)	8 (15.4)	8 (9.0)	
RAI therapy (%)			.379
Once	33 (63.5)	63 (70.8)	.207
Twice	15 (28.8)	15 (16.9)	
≥ 3	4 (7.7)	11 (12.4)	
RAI-refractory disease (%)	22 (42.3)	14 (15.7)	.001
Recurrence (%)	4 (7.7)	4 (4.5)	.467
All-cause mortality (%)	25 (48.1)	12 (13.5)	<.001
Disease-specific mortality (%)	14 (26.9)	6 (6.7)	.002

Values are absolute numbers (%), or mean (SD). All patients received a total thyroidectomy. Abbreviations: FTC, follicular thyroid carcinoma; IQR, interquartile range; OTC, oncocytic thyroid carcinoma; RAI, radioactive iodine.

^*a*^UICC/AJCC TNM 8th edition.

^*b*^Comparing OTC with FTC; *P* < .05 was considered significant.

### Reclassification of Tumors

**Figure 2.**
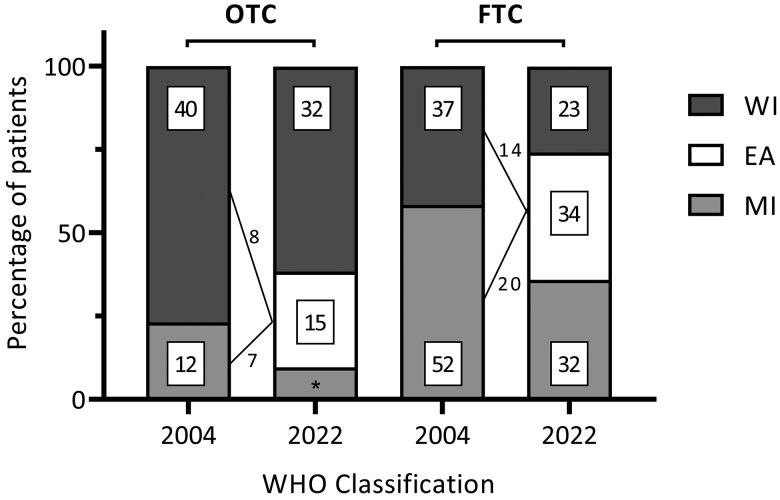
Reclassification of tumors according to the 2004 and 2022 WHO Classification. All values are absolute numbers; *n = 5.

Abbreviations: OTC, oncocytic thyroid carcinoma; FTC, follicular thyroid carcinoma; MI, minimally invasive; EA, encapsulated angio-invasive; WI, widely invasive.

Figure 2 depicts the tumor reclassification from the 2004 WHO Classification to the 2022 WHO Classification after histopathological revision of all tumors. In total, 15 (28.8%) OTC were reclassified, of which 7 shifted from MIOTC to EAOTC, and 8 tumors from WIOTC to EAOTC. This resulted in 5 MIOTC, 15 EAOTC, and 32 WIOTC. In FTC, 34 (38.2%) tumors were reclassified (20 from MI to EA and 14 from WI to EA), resulting in 32 MIFTC, 34 EAFTC, and 23 WIFTC.

In Table 2, the tumor characteristics stratified per histological subtype are shown for both OTC and FTC. Median tumor size was smallest in minimally invasive categories (MIFTC 28.0 mm [IQR, 21.5-47.5] and MIOTC 25.0 mm [IQR, 24-50]). A gradual increase in tumor size with each WHO category was observed for both OTC (EAOTC: 40.0 mm [IQR, 27.0-55.5] and WIOTC 45.0 mm [IQR, 35.3-63.8]) and FTC (EAFTC: 36.5 mm [IQR, 21.0-47.5] and WIFTC 40.mm [IQR, 33.0-71.0]). Three tumors exhibited no CI, but did have VI, and were therefore diagnosed as either EAFTC (n = 2) or EAOTC (n = 1). Furthermore, gross extra-thyroidal extension (OTC: n = 4 [12.5%], and FTC: n = 7 [30.4%]) and multinodular growth (OTC: n = 21 [67.7%], and FTC: n = 12 [52.2%]) were only observed in the WI subtypes.

**Table 2.** Patient and tumor characteristics per histological subtype according to the 2022 WHO Classification

	OTC			FTC		
	MI (n = 5)	EA (n = 15)	WI (n = 32)	MI (n = 32)	EA (n = 34)	WI (n = 23)
Male sex (%)	2 (40.0)	8 (53.3)	16 (50.0)	2 (6.2)	12 (35.3)	7 (30.4)
Age at diagnosis (years)	50.8 (5.1)	56.7 (18.9)	65.8 (10.6)	43.5 (14.2)	48.8 (18.8)	67.3 (11.1)
Lymph node metastasis present (%)	0 (0.0)	1 (6.7)	6 (18.8)	1 (3.1)	0 (0.0)	6 (26.1)
Distant metastasis present (%)	0 (0.0)	1 (6.7)	9 (28.1)	1 (3.1)	5 (14.7)	9 (39.1)
Tumor size (mm)	25.0 [24.0, 50.0]	40.0 [27.0, 55.5]	45.0 [35.3, 63.8]	28.0 [21.5, 47.5]	36.5 [21.0, 48.0]	40.0 [33.0, 71.0]
Extra-thyroidal extension (%)						
None	5 (100.0)	14 (93.3)	22 (68.8)	32 (100.0)	32 (94.1)	12 (52.2)
Microscopic	0 (0.0)	1 (6.7)	6 (18.8)	0 (0.0)	2 (5.9)	4 (17.4)
Gross	0 (0.0)	0 (0.0)	4 (12.5)	0 (0.0)	0 (0.0)	7 (30.4)
Multinodular growth pattern (%)	0 (0.0)	0 (0.0)	21 (67.7)	0 (0.0)	0 (0.0)	12 (52.2)
Capsular invasion*^a^* (%)						
0 foci	0 (0.0)	1 (6.7)	—	0 (0.0)	2 (5.9)	—
1-3 foci	3 (60.0)	6 (40.0)	—	24 (75.0)	15 (44.1)	—
≥ 4 foci	2 (40.0)	8 (53.3)	—	8 (25.0)	17 (50.0)	—
Vascular invasion (%)						
0 foci	5 (100.0)	0 (0.0)	3 (9.4)	32 (100.0)	0 (0.0)	3 (13.0)
1-3 foci	0 (0.0)	9 (60.0)	4 (12.5)	0 (0.0)	23 (67.6)	3 (13.0)
≥ 4 foci	0 (0.0)	6 (40.0)	25 (78.1)	0 (0.0)	11 (32.4)	17 (73.9)

Values are absolute numbers (%), mean (SD), or median (IQR). Abbreviations: EA, encapsulated angio-invasive; FTC, follicular thyroid carcinoma; IQR, interquartile range; MI, minimally invasive; OTC, oncocytic thyroid carcinoma; WI, widely invasive.

^*a*^In case of an encapsulated tumor.

### Effect on Clinical Outcomes

**Figure 3.**
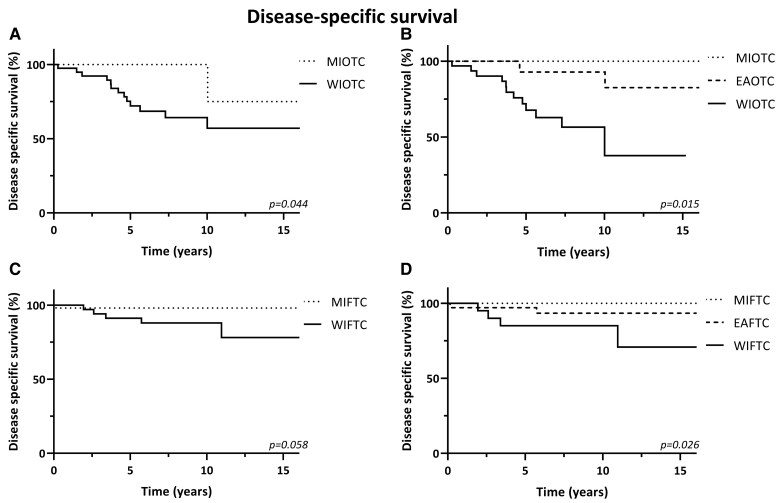
Kaplan-Meier curves for DSS in OTC and FTC, according to the 2004 and 2022 WHO Classification. A, 2004 WHO Classification in OTC; B, 2022 WHO Classification in OTC; C, 2004 WHO Classification in FTC; D, 2022 WHO Classification in FTC.

Figure 3 shows the Kaplan-Meier curves for DSS per WHO Classification for each histological subtype. The 2022 WHO Classification showed an improved risk stratification compared to the 2004 WHO Classification with an intermediate risk of disease-specific death for both EAOTC and EAFTC. The 10-year DSS rates in OTC following the 2022 WHO Classification were 100%, 92.9%, and 56.5% for MIOTC, EAOTC, and WIOTC (*P* = .015), respectively (see Fig. 3B), while these rates were 100% for MIOTC and 57.1% for WIOTC based on the 2004 WHO Classification (*P* = .044) (see Fig. 3A). Similar trends were observed for FTC, resulting in 10-year DSS rates of 100%, 93.3%, and 85% for MIFTC, EAFTC, and WIFTC, respectively (*P* = .026) (see Fig. 3D), and 98.1% and 87.9% for MIFTC and WIFTC following the 2004 WHO Classification (*P* = .058) (see Fig. 3C and Table 3).

**Table 3.** Ten-year rates per WHO Classification for each histological subtype

	Disease-specific survival*^a^*		RAI-refractory disease*^b^*		Overall survival*^a^*		Recurrence-free survival*^a^*	
	2004	2022	2004	2022	2004	2022	2004	2022
MIOTC	100%	100%	31.3%	25.0%	90.9%	80.0%	88.9%	80.0%
EAOTC	-	92.9%	-	41.2%	-	85.7%	-	90.0%
WIOTC	64.2%	56.5%	53.2%	52.9%	41.2%	29.0%	82.2%	82.5%
MIFTC	98.1%	100%	2.2%	5.1%	95.7%	100%	100%	100%
EAFTC	-	93.3%	-	6.3%	-	89.9%	-	96.0%
WIFTC	87.9%	85.0%	35.1%	50.3%	83.4%	77.3%	76.2%	64.3%

Abbreviations: EA, encapsulated angio-invasive; FTC, follicular thyroid carcinoma; MI, minimally invasive; OTC, oncocytic thyroid carcinoma; WI, widely invasive.

^*a*^10-year survival rate.

^*b*^10-year incidence rate.

**Figure 4.**
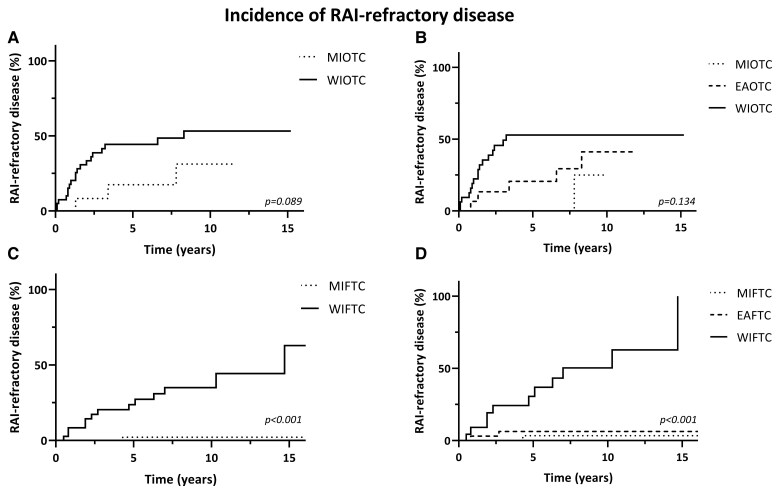
Kaplan-Meier curves for incidence of RAI-refractory disease in OTC and FTC, according to the 2004 and 2022 WHO Classification. A, 2004 WHO Classification in OTC; B, 2022 WHO Classification in OTC; C, 2004 WHO Classification in FTC; D, 2022 WHO Classification in FTC.

In Fig. 4, the Kaplan-Meier curves for the incidence of RAI-refractory disease are depicted. Comparing the 2022 to the 2004 WHO Classification, the EA subtype had an intermediate risk in terms of developing RAI-refractory disease in the OTC group (see Fig. 4A and 4B). For EAFTC, however, the risk of RAI-refractory disease was similar to MIFTC (see Fig. 4C and 4D). The 10-year incidence rates for the 2022 WHO Classification were 25%, 41.2%, and 52.9% for MIOTC, EAOTC, and WIOTC (*P* = .134), respectively. For MIFTC, EAFTC, and WIFTC, these rates were 5.1%, 6.3%, and 50.3% (*P* < .001) (see Table 3).

The Kaplan-Meier curves for OS and RFS are shown in Supplementary Figs. S1 and S2, with the corresponding 10-year survival rates listed in Table 3 (33). In patients that developed no evidence of disease (n = 29 OTC and n = 63 FTC), 4 (13.8%) OTC and 4 (6.3%) FTC patients developed recurrence of either structural or biochemical disease. For both OS and RFS, an intermediate survival was observed in EAFTC, whereas in OTC no clear improvement was seen with the 2022 WHO Classification compared to the 2004 WHO Classification.

## Discussion

To the best of our knowledge, this study is the first to investigate the impact of the reclassification by the 2022 WHO Classification compared to the 2004 WHO Classification on relevant clinical outcomes in OTC and FTC. We found that EAOTC and EAFTC had an intermediate prognosis in-between the MI and WI subtypes, especially for DSS and incidence of RAI-refractory disease. For OS and RFS, a similar intermediate prognosis was observed in EAFTC, but not in EAOTC.

In both OTC and FTC, a substantial number of tumors were reclassified to the EA subtype. Specifically, 7 MIOTC and 20 MIFTC changed to the EA category due to the presence of VI in (entirely) encapsulated lesions, resulting in a subgroup with a risk of disease-specific death in-between the MI and WI subtypes. These findings are consistent with earlier studies, which show that the presence and extent of VI are associated with lower DSS in FTC (9, 21, 22, 24, 34-36). Our findings also confirm this trend for OTC patients. Furthermore, 8 WIOTC and 14 WIFTC cases were reclassified to the EA category. This group of tumors consisted of (entirely) encapsulated lesions with VI and with or without CI to such an extent that the diagnosis of MI, as defined by the 2004 WHO Classification, did not suffice. In other words, the lesions exhibited ≥ 4 foci of CI in combination with VI but were still entirely encapsulated. As a result, this group was diagnosed as EAFTC or EAOTC instead of WIFTC or WIOTC following the 2022 WHO Classification (29). Tumors classified as WI are defined as lesions with widespread invasion through the thyroid; nevertheless, this category consists of a very heterogeneous group of tumors in terms of the extent of invasiveness. The consequence of this new definition, in our study, was a reclassification of a subgroup of WI patients who were less likely to die due to thyroid carcinoma than those without an encapsulated tumor as shown in Fig. 3. Future research should establish whether addition of Ki-67 immunohistochemistry to the currently used WHO definition could potentially improve the WHO Classification and further substantiate the diagnosis of WI tumors, as Ki-67 is associated with higher recurrence rates and lower DSS in FTC (37, 38). All in all, our results highlight the importance of accurate assessment of the tumor capsule and CI, in addition to VI, as entirely encapsulated tumors seem to have a lower risk of DSS among others compared with those without full encapsulation.

Our study shows that patients with EAOTC or EAFTC have an intermediate risk of disease-specific death, in-between the MI and WI subtypes. A recent study by Matsuura et al found a similar trend in a population of 79 FTC and 111 OTC patients with a median follow-up time of 82 months (IQR, 35-125), but they adopted a different approach (18). In that study, the EA subgroups were split according to extent of VI and combined with either MI or WI subtypes before analysis, due to the few WI cases in their series (1 WIOTC and 8 WIFTC). The 10-year DSS rates were 100% (MIFTC and EAFTC with focal VI), 86% (EAFTC with extensive VI and WIFTC), 96% (MIOTC and EAOTC with focal VI), and 77% (EAOTC with extensive VI and WIOTC). Differences in these survival rates compared to ours could potentially be explained by the mixing of histological subtypes, but also the higher number of patients presenting with distant metastasis in our cohort (1%-4% vs 17%-19% in this study). Furthermore, it should be noted that our series comprised more patients with WIFTC and WIOTC. A possible explanation for this difference in disease severity could be that, in the Netherlands, the use of neck ultrasound is less easily accessible and thyroid incidentalomas on computed tomography or magnetic resonance imaging have not been routinely investigated (32, 39). As a consequence, patients in the Netherlands generally present with a more advanced disease stage than in other high-income countries where neck ultrasounds are more easily accessible (39, 40). Another explanation is that the patients included were recruited from a tertiary university hospital, potentially leading to a more aggressive case mix of tumors and relatively large set of WI carcinomas.

For the incidence of RAI-refractory disease, an intermediate risk was observed in EAOTC patients, in-between MIOTC and WIOTC. In FTC, however, this difference was less clear with the MI and EA subtypes conferring a similar risk of developing RAI-refractory disease. As expected, RAI-refractory disease was more common in the OTC group than the FTC group, which aligns with earlier studies showing a tendency for OTC to be less RAI-avid than its FTC counterpart (41-43). However, our findings suggest that patients with MIOTC as defined by the 2022 WHO Classification without VI have a low risk of developing RAI-refractory disease, in contrast to the 2004 WHO Classification MI subtype with minor or without VI. Furthermore, our findings point out that especially in EAOTC and WIOTC patients, RAI-refractory disease is a relevant outcome to take into account during treatment. In contrast, specifically WIFTC tumors seem to be associated with RAI-refractory disease; however, the number of RAI-refractory FTC patients was relatively low, emphasizing the need to replicate our findings in a larger (multicenter) study to consolidate these results.

For OS and RFS, EAFTC showed an intermediate prognosis, in-between MIFTC and WIFTC, while the EAOTC subtype did not. Matsuura et al reported a lower OS and RFS for both OTC and FTC in the subgroups with the more aggressive histology (ie, EA subtypes with extensive VI and WI) (18). However, whether these rates are comparable to ours cannot easily be concluded due to the aforementioned differences in subgroups as well as the fact that a different definition for recurrence was used. A possible explanation for the different results between OTC and FTC could be the low numbers of recurrences and the sparse representation of MIOTC within our cohort, warranting cautious interpretation of the data.

A major strength of this study is the histopathological review performed independently by 2 pathologists to validate the diagnosis and critically assess the extent of invasiveness in a structured manner. Moreover, they were blinded for the outcome, which ensured a nonbiased approach to the revision. Another strength is the long follow-up time of 8.5 years, which enabled us to study multiple relevant clinical outcomes. Furthermore, this study also took RAI-refractory disease into account as an important clinical outcome, which has not been studied in this context before. A limitation of this study is the retrospective study design, and therefore, inevitably not all tissue could be retrieved from other hospitals. However, the Dutch Nationwide Pathology Database (Palga) enabled us to retrieve tissue from more than 90% of the initial patient cohort. Furthermore, although the sample size is relatively large given the rarity of both disease entities, we could not perform a Cox-regression analysis to study the relative risks for each outcome and correct for potential confounders. Preferably, this study should be replicated in a larger patient cohort to validate our results. However, performing a histopathological review generally is laborious and time-consuming, and can therefore limit the sample size for feasibility reasons. Another limitation could be referral and treatment bias, as all patients were recruited from a single tertiary university hospital. Possibly, this could have affected the study population, as the availability of advanced treatment modalities potentially attracted patients with more aggressive thyroid carcinoma. Lastly, it is known that a high interobserver variation exists in assessing capsular and vascular invasion in FTC, which can introduce relevant bias to the results (44). As such, 2 pathologists revised all tissues independent from each other and blinded to outcome, but any residual confounding cannot be ruled out.

In conclusion, classification of OTC and FTC into 3 subcategories based on the extent of invasiveness (ie, MI, EA, and WI), as defined by the 2022 WHO Classification, improved discrimination between low-, intermediate-, and high-risk patients, especially for DSS and incidence of RAI-refractory disease. Our findings emphasize accurate assessment of histopathological features such as VI, but also encapsulation and CI, in OTC and FTC to identify patients at higher risk of RAI-refractory disease and disease-specific death.

## Data Availability

The data that support the findings of this study are available from the corresponding author, W.E.V., upon reasonable request. The data are not publicly available due to privacy restrictions, as it contains information that could compromise the privacy of research patients.

## Abbreviations

CI: capsular invasion
DSS: disease-specific survival
DTC: differentiated thyroid carcinoma
EA: encapsulated angio-invasive
EAFTC: encapsulated angio-invasive follicular thyroid carcinoma
EAOTC: encapsulated angio-invasive oncocytic thyroid carcinoma
FTC: follicular thyroid carcinoma
IQR: interquartile range
MI: minimally invasive
OS: overall survival
OTC: oncocytic thyroid cancer
PTC: papillary thyroid cancer
RAI: radioactive iodine
RFS: recurrence-free survival
VI: vascular invasion
WI: widely invasive
